# Transforming primary healthcare by including the stakeholders involved in delivering care to people living in poverty: EQUIhealThY study protocol

**DOI:** 10.1186/1472-6963-13-92

**Published:** 2013-03-11

**Authors:** Christine Loignon, Catherine Hudon, Alexandrine Boudreault-Fournier, Sophie Dupéré, Ann C Macaulay, Pierre Pluye, Isabelle Gaboury, Jeannie L Haggerty, Martin Fortin, Émilie Goulet, Mireille Lambert, Luce Pelissier-Simard, Sophie Boyer, Marianne de Laat, Francine Lemire, Louise Champagne, Martin Lemieux

**Affiliations:** 1 Faculty of Medicine and Health Sciences, Department of Family Medicine, Université de Sherbrooke, Québec, Canada; 2 Department of Anthropology, University of Victoria, Victoria, British-Colombia, Canada; 3 Faculty of Nursing, Université Laval, Québec, Canada; 4 Participatory Research at McGill, McGill University, Québec, Canada; 5 Department of Family Medicine, McGill University, Québec, Canada; 6 Centre de santé et services sociaux du Chicoutimi, Québec, Canada; 7 ATD Fourth World Movement Canada, Québec, Canada; 8 The College of Family Physicians of Canada, Mississauga, ON, Canada; 9 Academic Primary Care Unit Charles-LeMoyne, Québec, Canada; 10 Academic Primary Care Unit Chicoutimi, Québec, Canada

**Keywords:** Participatory research, Poverty, Primary care, Photovoice, Knowledge transfer

## Abstract

**Background:**

Ensuring access to timely and appropriate primary healthcare for people living in poverty is an issue facing all countries, even those with universal healthcare systems. The transformation of healthcare practices and organization could be improved by involving key stakeholders from the community and the healthcare system in the development of research interventions. The aim of this project is to stimulate changes in healthcare organizations and practices by encouraging collaboration between care teams and people living in poverty. Our objectives are twofold: 1) to identify actions required to promote the adoption of professional practices oriented toward social competence in primary care teams; and 2) to examine factors that would encourage the inclusion of people living in poverty in the process of developing social competence in healthcare organizations.

**Methods/design:**

This study will use a participatory action research design applied in healthcare organizations. Participatory research is an increasingly recognized approach that is helpful for involving the people for whom the research results are intended. Our research team consists of 19 non-academic researchers, 11 academic researchers and six partners. A steering committee composed of academic researchers and stakeholders will have a decision-making role at each step, including knowledge dissemination and recommendations for new interventions. In this project we will adopt a multiphase approach and will use a variety of methods, including photovoice, group discussions and interviews.

**Discussion:**

The proposed study will be one of only a few using participatory research in primary care to foster changes aimed at enhancing quality and access to care for people living in poverty. To our knowledge this will be the first study to use photovoice in healthcare organizations to promote new interventions. Our project includes partners who are targeted for practice changes and improvements in delivering primary care to persons living in poverty. By involving knowledge users, including service recipients, our study is more likely to produce a transformation of professional practices and encourage healthcare organizations to take into account the needs of persons living in poverty.

## Background

According to the United Nations Committee on Economic, Social and Cultural Rights, poverty is “a human condition characterized by sustained or chronic deprivation of the resources, capabilities, choices, security and power necessary for the enjoyment of an adequate standard of living and other civil, cultural, economic, political and social rights.” [1] This definition includes the dimensions of power and dignity, which are relevant to the study of provision of care that is responsive to the needs and social conditions of people living in poverty (PLPs), i.e., socially responsive care. In Canada, poverty primarily affects women, especially single mothers, as well as immigrants, First Nations peoples, and adults between the ages of 45 and 64 years living alone [2]. Poverty and health inequities are major issues worldwide, and in many countries, undertaking intersectoral action to diminish the social gradient is on healthcare decision-makers’ agenda [3].

Living in poverty increases the risk of developing a chronic illness [4,5]. PLPs are at greater risk for chronic illnesses, deterioration in health status, and premature death [6-8]. Despite this reality, they are the ones least well served in matters of healthcare services (inverse care law) [9]. PLPs have less access to family physicians [10] and report having healthcare needs that are less often satisfied, as compared to people with higher incomes [11]. Finally, their experiences of healthcare services are more often negative, and sometimes they feel judged by the professionals providing their care [11-15].

Healthcare system changes and the cost of certain health services can constitute major burdens for some patients. Faced with repeated reforms and the trend toward privatization of healthcare services, some users, particularly those without a family physician, do not know where to obtain care and are increasingly obliged to pay for services [16]. According to our recent study (Loignon and Haggerty, 2012), both indirect and direct costs of healthcare discourage the poorest from using healthcare services. People with the lowest incomes pay proportionately more for healthcare-related costs than do those who are better off [17].

Individuals living in poverty can experience psychological suffering, numerous physical ailments and problems of social exclusion, even when they belong to a social support network. Living in poverty subjects people to detrimental conditions such as food insecurity, stress, inadequate housing, isolation, and experiences of violence and discrimination. These living conditions constitute major obstacles to self-management of chronic illnesses [13,15,18,19]. When compounded by difficulties in accessing and using primary care services, these obstacles perpetuate ill health among PLPs.

Studies have shown that low-income patients have more difficulty managing treatments and consult less often for preventive services, compared to those with higher incomes [13,18,20]. Low-income patients are more at risk of discontinuing care [21]. They may have trouble understanding medical advice and biomedical terminology, and they report less satisfying experiences of care and unmet health needs [22-24]. These people are especially sensitive to attitudes and to what their physician tells them in clinical visits [25]. They feel stigmatized because of their social status and perceive a lack of sensitivity among professionals with regard to their living conditions, and this has a negative impact on their use of healthcare [13,19,26,27]. Those receiving employment assistance benefits may experience a sense of devaluation when coming in contact with workers in the health and social services sector because they belong to a social group that is the subject of prejudice [28].

Health professionals also face major challenges in dealing with poor patients who have coexisting chronic illnesses [28,29]. These persons tend to adopt a more passive style of communication, talk less, ask fewer questions, and especially, take a less active role in the process of choosing treatments, making it difficult to establish a therapeutic physician–patient alliance [30]. This has repercussions on healthcare interactions, since physicians devote less time, provide less information, and do less reinforcement of treatment compliance with these patients [24,31]. Because they have a poor understanding of the social situations of their patients living in poverty, they become frustrated, which then leads them to develop a negative attitude toward these patients [30,32]. Health professionals play an important role in the daily lives of the persons they treat [33]. Therefore, it is important for PLPs to receive socially responsive care [9,15], because the quality of the patient–professional relationship is a key factor in the effectiveness of care [13,25,34]. Professionals’ poor understanding of their patients’ social conditions, prejudices regarding poverty, and inadequate training may compromise the quality of clinical interactions [30,35].

### Developing social competence in health care organizations

Data from two of our recent studies (Loignon et al., 2009) indicate that physicians providing care in settings of poverty have developed knowledge, skills, and strategies that support effective care interactions, in spite of the social distance between them and PLPs [5]. Our studies revealed that physicians’ development of social competence is promoted by close interprofessional collaboration—or team cohesion—that is sustained over time and directed toward the needs of PLPs. Given that our previous studies focused on approaches adopted by physicians in urban settings of poverty whose expertise has developed over the years, we decided to expand the study of social competence to the entire primary care team by involving different health professionals and academic healthcare organizations. In addition, we believe it is imperative at this stage to include PLPs in the search for solutions that would promote social competence not only in health professionals, but also in healthcare organizations. Incorporating PLPs into the process of research in primary care is a promising avenue that, to date, has received little attention.

Even in the context of participatory research in health, which historically has supported the involvement of vulnerable persons, there is room for improvement. In fact, vulnerable patients’ involvement in the development and organization of primary healthcare delivery is minimal. We recently performed a literature review on the role of vulnerable groups (i.e., persons living in poverty or experiencing barriers to care and living with chronic conditions) in primary healthcare (Charlebois K, Loignon C, Boudreault-Fournier A, Dupéré S, Grabovschi C: L’implication des personnes vulnérable dans la recherche participative en soins primaires: une revue de littérature, submitted). Our review raised important concerns and demonstrated that the level and quality of those vulnerable groups’ involvement were paradoxically jeopardized even when the projects analyzed used a participatory approach. For instance, in the 33 participatory research projects we reviewed, vulnerable groups were included in data analysis and knowledge transfer activities in only 21% and 15% of the projects, respectively.

### Research objectives

The aim of this innovative participatory action research project is to stimulate PLP-supportive changes in healthcare organizations and practices by encouraging collaboration between care teams and PLPs. Our objectives are: a) to identify actions required to promote the adoption of professional practices oriented toward social competence in primary care teams; and b) to examine factors that would encourage the involvement of PLPs in the process of developing social competence in healthcare organizations.

## Methods/design

This project is based on a participatory action research design applied in a clinical setting. Participatory research is an increasingly recognized methodological approach that is helpful for involving the people for whom the research results are intended [36]. Israel et al. (1998) defined participatory research as a research approach that recognizes the socially constructed nature of scientific knowledge [37]. Participatory action research is differentiated also by the involvement of researchers from outside the academic setting. These non-academic researchers—members of the community or representatives of organizations—participate in all stages of the research. This equitable participation, based on a collaborative approach among the partners, allows the non-academic members to benefit immediately from the research findings or to become involved in knowledge transfer [38,39]. The participatory research process adopted for the EQUIhealThY project is based on an approach developed by ATD (All Together in Dignity) Fourth World Movement, our main partner from the non-scientific community. (This organization, active in some 30 countries, transcends national boundaries and is concerned with the welfare of the most vulnerable at the international, national and locals levels.) Their approach, called the “merging of knowledge and practice”, is one they have tested in Europe in similar initiatives; it enables free and open discussion that encourages the sharing of different views as well as the involvement of PLPs in the research process [40]. A fundamental operating principle of this method is that, first, people are supported in reflecting on issues with their peer groups, i.e., the professionals among themselves and PLPs also among themselves, separately, with the help of trained facilitators. In our study, PLPs will be supported by volunteers from ATD Fourth World Movement. This approach will give each peer group the time they need to reflect and to prepare for interactions with the others. It also will help ensure that PLPs’ full participation is not inhibited by unequal power relationships. As well, with a small group there is a greater likelihood that participants’ involvement will be facilitated and that the project will succeed [38,41].

As recommended by participatory action research guidelines, a charter of guiding principles will be adopted during the first months of the project. The purpose of this charter will be to define the objectives and the terms of the participatory component of the research, including participation, consent, access to data, and dissemination of results. The principles in the charter will be specified and adopted by all the partners. The process is thus based on collaboration and consensus, since all the partners are considered to be researchers and all are equal within the context of this research project. To achieve this aim, regular meetings will be held, particularly with the volunteers from ATD Fourth World Movement, to strengthen collaboration among the partners.

Our project will also use the *photovoice* method. Photovoice is a participatory research method that has been used in several healthcare research projects [42]. However, to date it has not been much used in primary care research with health professionals. This method “enable[s] participants to use their photographs to elicit emotions, feelings, and insights about topics that may be shrouded in silence” (p. 376) [42]. Like other methods, such as interviews and focus groups, photovoice is a means of generating knowledge on the lived experience of researcher-subjects [43]. This method offers the advantages of generating introspection and knowledge acquisition, while promoting critical dialogue [43,44].

### Participants and sampling

Our project will be carried out simultaneously in two primary care settings that have a dual mission: a *population*-*focused* mission of responding to the needs of the population in their territory in accordance with their status as family medicine groups in Quebec, and an *academic* mission to train family medicine residents, in line with their status as family medicine units. These two primary care organizations serve very different populations in terms of care experiences and poverty, which is a great asset for the study. The first clinic is in an urban setting, and a portion of its clientele consists of people on social assistance, people with substance dependencies, and immigrants living in poverty. The second clinic is in an urban setting far removed from the large centres, and serves a varied clientele that includes poor workers, people on social assistance, and some first-generation immigrants (Table 1).

**Table 1:** Knowledge-user partners

**Knowledge**-**user partners**	**Organization**	**Role**
Sophie Boyer/Marianne de Laat	ATD Fourth World Movement [international anti-poverty organization]	Volunteers
Luce Pélissier-Simard	Faculty of Medicine, University of Sherbrooke	Director of the family medicine residency program at the University of Sherbrooke
Francine Lemire	The College of Family Physicians of Canada (CFPC) [federal-level professional organization responsible for the training of family physicians in Canada]	Executive Director and Chief Executive Officer
Louise Champagne	Academic Primary Care Unit Charles-LeMoyne	Director of the APCU in Saint-Lambert, Quebec
Martin Lemieux	Academic Primary Care Unit Chicoutimi	Director of the APCU in Chicoutimi, Quebec

**Figure 1:**
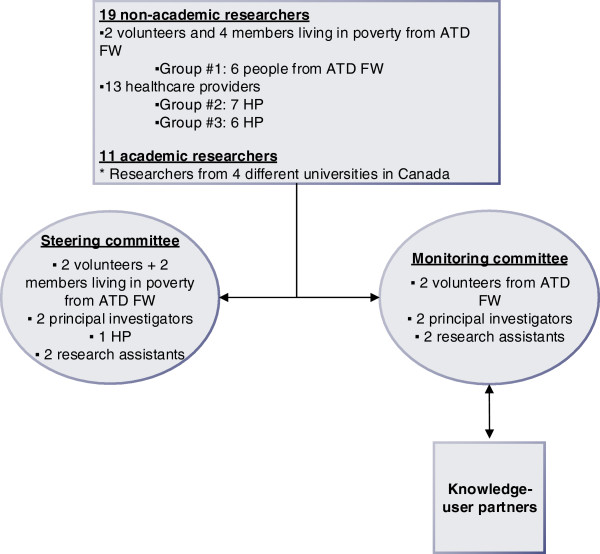
Participants and governance structure.

Our research team consists of 19 non-academic researchers, 11 academic researchers and six partners. The participatory approach is based on the involvement of healthcare professionals and members of ATD Fourth World Movement in three groups. The first group (Group 1) is made up of members of ATD Fourth World Movement and consists of four PLPs with at least two chronic illnesses and two volunteers. Groups 2 and 3 are made up of seven and six health professionals respectively from two academic primary care units (APCU): 3 physicians, 3 nurses, 1 receptionist, 4 residents, 1 psychologist and 1 social worker (Figure 1).

The steering committee, already constituted, is made up of members from each of these groups, i.e., researchers, PLPs, ATD Fourth World Movement volunteers and health professionals. The steering committee ensures the different actors are represented throughout the project’s entire process, and it has a decision-making role. More specifically, it is the steering committee that will select the research questions and the materials (photos, narratives) that will be analyzed; the committee will develop the analysis tools, and participate in the data analysis and in developing the knowledge dissemination plan.

### Data collection

This project will be carried out in four phases spread over three years.

#### Phase 1: Photovoice

The first phase will be devoted to exploring a research question in small, separate groups using the photovoice method. Each group involved in this project, i.e., the PLPs (Group 1) and the health professionals (Groups 2 and 3), will undergo training in the photovoice method, take photos in the community, and attend photovoice meetings and a merging of knowledge and practice meeting, where the health professionals and the PLPs will present their photos to each other and discuss the results.

The three groups will respond to the research question: What are the barriers between the healthcare team and PLPs? This research question has been decided on by the steering committee. The PLPs, health professionals and researchers will each be asked individually to take photos that, in their view, represent poverty and illustrate the consequences for the experience of care. Three to four weeks will be allotted to taking photos. Each participant will be invited to select five photos from all the photos taken during this period. These will be used to stimulate reflection and help give all participants a voice. First, the participants will each present their photos in their own group, for discussion. The members of each group will then select three to five images that respond to the research question and that will be presented to the other groups. The meetings between peer groups involving professionals will be recorded and transcribed. Subsequently, the PLP group will meet with the groups of professionals for “merging of knowledge and practice”, a method developed by ATD Fourth World Movement. The groups will discuss the photos that they have selected to present to each other. These knowledge and practice merging sessions will also be recorded and transcribed. After these meetings, the steering committee will produce a coding grid and an analysis grid. The steering committee members will take part in the analysis of the merging knowledge meetings. To support this process, the academic researchers will provide a training session in qualitative analysis for the non-academic researchers. The research assistants will code the transcripts and discussions and will prepare summary tables of the data. The steering committee will validate the interpretation of the analyses of the data obtained.

#### Phase 2: Interaction narratives

In a second phase, we will invite the three groups to reflect on and share their mutual expectations regarding the professionals’ role toward PLPs. We will invite the three groups to reflect independently on their expectations of each other, and then to respond to the question: What is the role of health professionals in relation to PLPs? Or, more specifically, for a PLP, what constitutes a competent professional? Each group will meet to respond in their own way, and from their own perspective, to this question, which participants will be free to fine-tune as they see fit. In this way, we will gather their perceptions about the role of health professionals toward PLPs. The participants in each group will draft a short narrative describing an interaction between a professional and a PLP. Each group will discuss the narratives drafted and will draw up a list of important points raised by the whole group. This half-day meeting will be moderated by a facilitator in each group who will draft a summary of the narratives and will validate the content with the participants.

After the narratives have been written, the steering committee will select those that will be analyzed. Each group will analyze and interpret the selected narratives in order to respond to the research question. Finally, the PLP group will meet with the groups of professionals in an activity to merge knowledge and practice, at which all the selected narratives will be discussed and analyzed. They will identify divergences and similarities between the narratives and will draw up a list of concrete means of encouraging the development of social competence in primary care organizations.

The final step in the second phase will be the analysis of the discussion on the narratives and of the knowledge merging exercise. The data analysis procedure described in Phase 1 will also be followed in Phase 2.

#### Phase 3: Dissemination and presentation of results

Phase 3 will be devoted to the presentation of results. At the beginning of the project, we will invite all the participants to take part in developing a knowledge dissemination plan. This plan will be revised in Phase 3 as needed. We have already identified certain settings—community, professional, decision-making and academic—that are involved in providing care to PLPs, and where the dissemination of this project’s results would be relevant.

#### Phase 4: Implementation of actions to encourage the development of social competence in clinical settings

Phase 4 will support the implementation of actions to encourage the development of social competence in clinical settings. Our team will document the implementation of the actions adopted at each of the sites by means of semi-structured interviews carried out with managers of the family medicine units (director, medical services chief, etc.) and some (5 to 10) professionals. Individual interviews (5 to 10) may also be carried out with PLPs attending the clinic to assess their levels of perceived satisfaction. Altogether, a maximum of 20 interviews of about an hour each will be used to evaluate the degree of implementation of measures applied in the clinical settings. An interview guide will be developed by the research team, and the interviews will be recorded, transcribed and coded using NVivo software. Data analysis will consist of data reduction, results presentation, and development and validation of interpretations.

### Data analysis and interpretation

We will use a thematic analysis strategy to analyze the material. We will involve the non-academic researchers in analyzing the data from the merging of knowledge meetings. Indeed, the steering committee, made up of at least one representative of each group of non-academic researchers, will be involved in developing the coding grid and in analyzing and validating the interpretation of the data. The peer group meeting between the professionals’ groups will be analyzed by the research assistant and two researchers. They will do the data reduction and organization using NVivo software (QSR) [45]. We will develop a summary list of codes corresponding to the different themes addressed. This list will be modified over the course of the analysis as new codes are generated and pre-existing codes refined. The results will then be presented in tables summarizing the data obtained from each meeting or interview. These tables will also be used by the whole research team (researchers and collaborators) to develop and verify the conclusions.

Triangulation procedures will be used at every step of the study to validate the analyses and interpretations. The coding will be verified using double coding techniques, both inter-coder and intra-coder. Inter-coder double coding will be done primarily by the research assistant and a researcher, who will code each transcript in parallel and independently and then will compare their results. When there are discrepancies, they will clarify their differences, refine the codes, and re-do the coding [45]. Triangulation will also be applied to forming hypotheses and developing conclusions. Finally, we will conduct the study in accordance with the transferability criteria that characterize a qualitative research approach [46]. In fact, the detailed description of each stage of our study will allow the results obtained to be transferred and applied to other similar contexts [47].

### Ethical considerations

This study has been approved by the Research and Ethics Committees of both the Champlain-Charles-Lemoyne CSSS (health and social services centre) and the Chicoutimi CSSS. It is based on current ethical principles, such as each person’s freedom to refuse to participate in the study and to withdraw at any time, and respect for the participants. The adoption by consensus of a charter at the start of the project will promote mutual trust among the academic and non-academic researchers and will reinforce their understanding of the research process. We will guarantee, for the participants, strict confidentiality of records, and we will maintain the confidentiality of all statements by identifying each participant by number. The files and audiorecordings will be kept in a sealed location at the Research Centre of the Champlain-Charles-Lemoyne CSSS and will be destroyed after five years. No name will appear on any public documents and every precaution will be taken to ensure no information will be divulged that could allow participants to be identified by a third party. These ethical principles are clearly stated on the consent form signed by all the non-academic researchers.

## Discussion

EQUIhealThY is the third project in an extensive research program whose objective is to improve primary healthcare for persons living in poverty who have chronic illnesses. This participatory action research project, applied in clinical settings, is aimed at promoting collaboration between health professionals, on one hand, and PLPs and community organizations, on the other [48,49]. The two previous projects in this program generated data that supported the value of bringing care teams and PLPs together in a research project on the development of social competence in primary care.

Few participatory research projects are being undertaken with care teams in primary care organizations. An advantage of this study is that our methodological approach includes the adoption of a preliminary knowledge dissemination plan to be confirmed by all the researchers, both academic and non-academic, as well as by the partners. This plan will be reviewed and revised, as needed, in Phase 3, once the results of the study are clearly determined. Our project includes partners targeted for practice changes and improvements in the delivery of primary care to PLPs. By involving knowledge users, our study is more likely to produce a transformation of professional practices and encourage healthcare organizations to take into account the needs of PLPs.

It is important here to discuss the modulations that characterize participatory research design. Our protocol has been modified since the first version, reflecting our ongoing commitment to scientific quality and rigour. In fact, the reflexive quality of our project is based on the findings of a recent review of participatory research studies by Jagosh et al. (2012) [51]. They suggest that the adaptation of protocol procedures constitutes a form of quality control. They identified seven outcomes of effective participatory research studies that could serve as criteria for appraising the quality of such projects. In summary, effective participatory research projects will: 1) ensure culturally and logistically appropriate research; 2) enhance recruitment capacity; 3) generate professional capacity and competence in stakeholder groups; 4) result in productive conflicts followed by useful negotiation; 5) increase the quality of outputs and outcomes over time; 6) increase sustainability of project goals beyond funded time frames and during gaps in external funding; 7) create system changes and new unanticipated projects and activities.

We consider that our research project satisfies all these outcomes criteria, except for 5 and 6, which can, by their nature, only be assessed after some time, well beyond the end of the funding time frame. With regard to item 3, we believe the researchers will gain knowledge on the lived experience of poverty by working in close partnership with PLPs and through expertise acquired by volunteers from ATD Fourth World Movement. Also, the latter will gain experience in research and acquire specific skills in qualitative data analysis because they will be trained by researchers in data analysis and will be involved in that process. Another illustration of the quality of our research is related to criterion 4. In fact, we have already encountered disagreement and mutual misunderstanding at one point. We overcame those by increasing face-to-face and telephone meetings and by creating a monitoring committee. Our commitment to the principles of participatory research quality was also expressed in our decision to invite a researcher experienced in participatory research to act as a ‘key facilitator’ in helping us resolve these partnership misunderstandings. The ultimate outcome of this experience was a more resilient and committed partnership, which we consider to be a sign of quality within the framework outlines by Jagosh et al [50].

In conclusion, our data will be used to promote the adoption of professional practices oriented toward social competence in primary care teams and to encourage the involvement of PLPs. They could also be used to improve the training of future physicians and nurses, and to develop tools for healthcare managers and decision-makers. Our academic and professional partners will be involved in the dissemination of results. Our community partners will be involved in disseminating knowledge to PLPs and community organizations working with PLPs. We will write articles for publication in local media and will participate in citizen forums. We envision organizing an exhibit of photos taken by the project’s participants. We could also work with participating PLPs to prepare brief bulletins for PLPs with information and tools to help them navigate the healthcare system (for example, where to consult, how to prepare for a healthcare appointment, etc.)

## Abbreviations

PLP: People living in poverty; APCU: Academic primary care units

## Competing interests

The authors declare that they have no competing interests.

## Authors’ contributions

All authors agreed on the publication of this protocol paper. CL and CH prepared and edited the paper with the assistance of EG. All the co-authors participated in writing the protocol. They also read and approved the final version of the manuscript.

## Pre-publication history

The pre-publication history for this paper can be accessed here:

http://www.biomedcentral.com/1472-6963/13/92/prepub
